# Goldberg-Shprintzen Megacolon Syndrome Diagnosed in the Neonatal Period: A Case Report With Molecular Confirmation

**DOI:** 10.7759/cureus.108193

**Published:** 2026-05-03

**Authors:** Eleni Papaioannou, Efrosyni Anastasiadou

**Affiliations:** 1 Department of Neonatology, Neonatal Intensive Care Unit (NICU), Hippokration General Hospital of Thessaloniki, Thessaloniki, GRC

**Keywords:** corpus callosum hypoplasia, genetic counseling, genetic diagnosis, goldberg-shprintzen syndrome, hirschsprung disease, kifbp, microcephaly, neonatal diagnosis, neonatal intestinal obstruction

## Abstract

Goldberg-Shprintzen megacolon syndrome (GOSHS) is a rare autosomal recessive neurodevelopmental disorder characterized by Hirschsprung disease, microcephaly, neurodevelopmental impairment, and craniofacial dysmorphism.

We report a male neonate born at 35+4 weeks of gestation who presented on day 4 of life with abdominal distension and delayed passage of meconium. Clinical examination revealed microcephaly, generalized hypotonia, craniosynostosis, and dysmorphic facial features, with a positive family history of GOSHS. Hirschsprung disease was confirmed by rectal biopsy, and cranial imaging demonstrated structural brain abnormalities, including hypoplasia of the corpus callosum, and confirmed the presence of craniosynostosis. Targeted molecular analysis of the Kinesin Family Binding Protein (KIFBP) gene using polymerase chain reaction (PCR) amplification followed by Sanger sequencing identified compound heterozygous pathogenic variants, confirming the diagnosis during the neonatal period.

GOSHS should be considered in neonates presenting with Hirschsprung disease in combination with hypotonia, dysmorphic features, or neuroimaging abnormalities. Early molecular diagnosis may facilitate timely multidisciplinary management and genetic counseling.

## Introduction

Goldberg-Shprintzen megacolon syndrome (GOSHS; OMIM #609460) is a rare autosomal recessive neurodevelopmental disorder characterized by the association of Hirschsprung disease, neurodevelopmental impairment, and distinctive craniofacial features [1-8]. The syndrome was first described in 1981 by Goldberg and Shprintzen in siblings presenting with aganglionic megacolon and craniofacial anomalies [1]. Subsequent reports expanded the phenotype to include microcephaly, intellectual disability, epilepsy, and structural abnormalities of the central nervous system [3-14].

The molecular basis of GOSHS has been attributed to biallelic loss-of-function variants in the Kinesin Family Binding Protein (KIFBP) gene (formerly KIAA1279), which encodes a protein involved in cytoskeletal organization, microtubule dynamics, and intracellular transport. KIFBP plays a critical role in neuronal migration and axonal growth by regulating kinesin-mediated transport along microtubules; its dysfunction disrupts the development of both the enteric and central nervous systems, explaining the coexistence of aganglionosis in Hirschsprung disease and the associated neurological abnormalities in affected individuals [2]. Recent studies have demonstrated that KIFBP modulates kinesin motor activity and prevents aberrant microtubule binding, thereby influencing intracellular transport dynamics [15].

The condition is extremely rare, with an estimated prevalence of less than one per 1,000,000 individuals, and has been reported in a limited number of cases worldwide. Despite its congenital origin, GOSHS is infrequently diagnosed in the neonatal period [7,8]. In most reported cases, the diagnosis is established beyond the neonatal period, typically during infancy or early childhood, when gastrointestinal and neurological manifestations become more apparent [3-7,9-11]. Early identification of a syndromic etiology is clinically relevant, as it facilitates timely genetic counseling and guides multidisciplinary management [3,7,10].

We report a neonate with GOSHS diagnosed in the neonatal period, with the molecular confirmation of compound heterozygous pathogenic variants in the KIFBP gene. This case highlights the importance of early syndromic recognition in neonates presenting with Hirschsprung disease and associated neurological or dysmorphic features. To the best of our knowledge, this represents a rare neonatal presentation of GOSHS with molecular confirmation, further supported by familial genetic findings.

## Case presentation

We present a male neonate born to a gravida 4 mother by cesarean section at 35+4 weeks of gestation due to a previous cesarean delivery and spontaneous onset of labor. He was admitted to our neonatal intensive care unit on the fourth day of life, following delayed passage of meconium and progressive abdominal distention since birth. Physical examination revealed microcephaly (head circumference below the third percentile), generalized hypotonia, craniosynostosis, and distinctive facial dysmorphism, including a broad nasal bridge, hypertelorism, and low-set ears. Neurological examination was notable for reduced spontaneous movements and generalized hypotonia, with preserved primitive reflexes and no focal neurological deficits identified. Key neonatal clinical findings at admission are summarized in Table 1.

**Table 1:** Neonatal clinical data at admission

Parameter	Finding
Gestational age	35+4 weeks
Apgar score	8 at one minute; 9 at five minutes
Birth weight (centile)	2.42 kg (10th to 50th)
Head circumference (centile)	29 cm (<3rd)
Length (centile)	47 cm (50th)
Age at admission	Day 4 of life
Presenting symptoms	Delayed meconium passage, abdominal distension
Neurological findings	Generalized hypotonia
Dysmorphic features	Broad nasal bridge, hypertelorism, low-set ears
Abdominal imaging	Distended loops
Neuroimaging findings	Corpus callosum hypoplasia
Cranial imaging (CT)	Craniosynostosis

Family history was notable for parents who were heterozygous carriers of different KIFBP variants. The first child had previously been diagnosed with GOSHS, with molecular confirmation by whole exome sequencing identifying pathogenic variants in the KIFBP gene, while the other two siblings were healthy.

**Figure 1:**
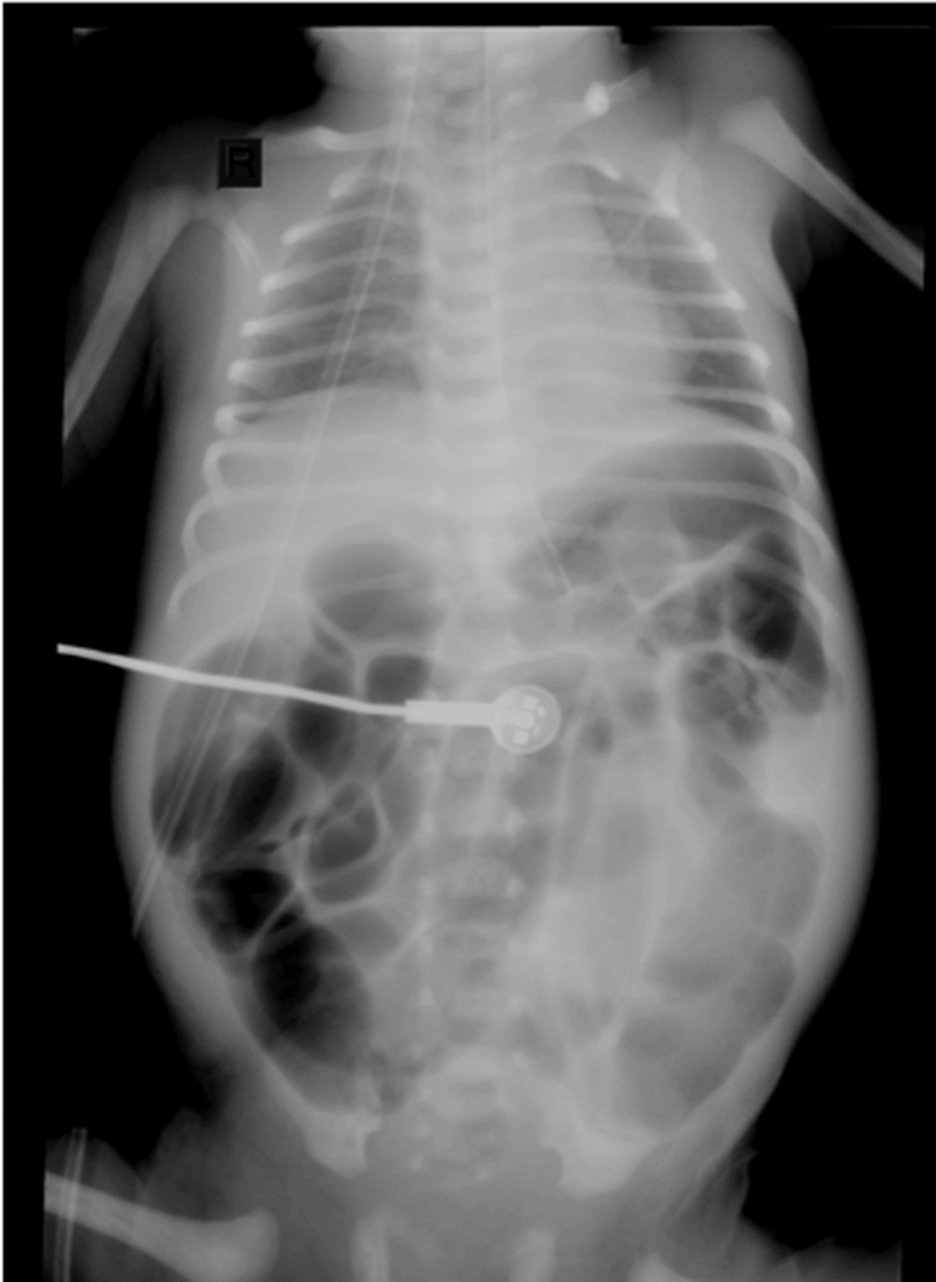
Abdominal X-ray demonstrating diffuse distension of intestinal loops, consistent with distal bowel obstruction in the context of Hirschsprung disease

The initial diagnostic evaluation was based on abdominal imaging. Abdominal X-ray imaging showed distended intestinal loops (Figure 1), consistent with distal bowel obstruction in the context of Hirschsprung disease. The neonate underwent surgical management. Colostomy was performed, and rectal biopsy confirmed Hirschsprung disease.

The combination of neonatal intestinal obstruction, generalized hypotonia, craniofacial dysmorphism, and a positive family history raised early clinical suspicion for an underlying syndromic condition, prompting targeted genetic evaluation.

**Figure 2:**
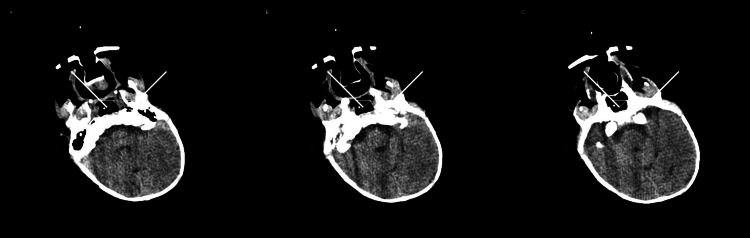
Cranial computed tomography demonstrating craniosynostosis involving the sphenoid and temporal bones bilaterally (arrows)

Cranial ultrasonography revealed hypoplasia of the corpus callosum. Computed tomography of the skull demonstrated craniosynostosis between the sphenoid bone and the squamous portion of the temporal bone bilaterally, as well as between the squamous and petrous portions of the temporal bone bilaterally (Figure 2), supporting a syndromic etiology involving cranial suture abnormalities.

Genetic findings

Targeted molecular genetic analysis was performed on peripheral blood DNA. The coding regions and exon-intron boundaries of the KIFBP gene (NM_015634.4) were analyzed using polymerase chain reaction (PCR) amplification followed by Sanger sequencing. Sequence analysis identified two heterozygous truncating variants, namely, c.1176_1177del (p.Glu394Thrfs*11) and c.1535C>G (p.Ser512*), consistent with compound heterozygosity. Both variants were classified as pathogenic according to the American College of Medical Genetics and Genomics (ACMG) criteria (PVS1, PM2, PP3). Familial genetic analysis confirmed segregation of the variants in trans, supporting autosomal recessive inheritance.

In the affected sibling, whole exome sequencing had previously identified the same pathogenic variants, which were subsequently confirmed by targeted Sanger sequencing, providing independent molecular validation of the diagnosis. The genetic findings are summarized in Table 2.

**Table 2:** Genetic findings KIFBP: Kinesin Family Binding Protein; PCR: polymerase chain reaction; ACMG: American College of Medical Genetics and Genomics

Parameter	Finding
Gene analyzed	KIFBP
Reference transcript	NM_015634.4
Method	PCR amplification and Sanger sequencing
Variants	c.1176_1177del (p.Glu394Thrfs*11); c.1535C>G (p.Ser512*)
Zygosity	Compound heterozygous
ACMG classification	Pathogenic (PVS1, PM2, PP3)
Segregation analysis	Variants confirmed in trans in both parents

## Discussion

Overview of GOSHS

GOSHS is an extremely rare disorder, with a limited number of molecularly confirmed cases reported in the literature to date [7,12,13]. The condition follows an autosomal recessive inheritance pattern and is caused by biallelic pathogenic variants in the KIFBP gene, most commonly truncating variants consistent with loss of function. The encoded protein plays a crucial role in axonal transport, cytoskeletal organization, and neuronal migration [2,7,11,12]. Recent experimental studies have further demonstrated that KIFBP regulates kinesin motor activity and modulates microtubule interactions, thereby influencing intracellular transport dynamics [15].

Pathophysiologically, loss of KIFBP function disrupts the development of both the enteric nervous system and the central nervous system, accounting for the hallmark combination of Hirschsprung disease, neurodevelopmental impairment, and brain malformations such as pachygyria and cerebellar hypoplasia. Disruption of KIFBP-mediated axonal transport has also been implicated in neurodevelopmental disorders, providing a mechanistic framework for the neurological manifestations observed in affected individuals [16]. Further evidence also suggests involvement of the peripheral nervous system, with several reports describing axonal neuropathy [2,5,8,12]. Experimental data further support that loss of KIFBP function disrupts cytoskeletal organization and intracellular transport, contributing to variability in neurological phenotypes [15,16].

Although congenital in origin, the timing of diagnosis is highly variable. While the earliest manifestations may appear in the neonatal period, additional neurological and developmental abnormalities often become apparent later in infancy or childhood, suggestive of phenotypic heterogeneity [3-7,9-11]. In the present case, the coexistence of Hirschsprung disease, generalized hypotonia, microcephaly, and corpus callosum hypoplasia is consistent with the core phenotypic spectrum described in previously reported cohorts. The presence of generalized hypotonia may reflect the early impairment of neuronal function related to disrupted axonal transport mechanisms. The early neonatal presentation further highlights the variability in the timing of diagnosis.

Review of published cases

A narrative review of the literature was conducted using published case reports and case series identified through PubMed and related databases, focusing on reports describing neonatal presentation, timing of diagnosis, and molecular confirmation of GOSHS. Gastrointestinal manifestations, particularly Hirschsprung disease or severe intestinal dysmotility, are present in most reported cases [1-6,8-10,14]. Neurological features include global developmental delay, intellectual disability, epilepsy, hypotonia, and structural brain abnormalities [2-6,9,14]. Craniofacial dysmorphism, often subtle in the neonatal period, commonly includes microcephaly, hypertelorism, thickened lower vermilion, broad nasal bridge, thick eyebrows, and low-set ears, which often become more apparent with progressive age [3-6]. Although craniofacial dysmorphism is commonly reported, craniosynostosis is not a typical feature of GOSHS and has only rarely been described in the literature. Structural brain abnormalities, such as agenesis or hypoplasia of the corpus callosum, ventriculomegaly, and cerebellar hypoplasia, have been detected on early neuroimaging in some neonates, while cortical malformations, including polymicrogyria, are well documented in older children and adolescents [4-6,9]. In the present case, neuroimaging demonstrated hypoplasia of the corpus callosum on cranial ultrasonography and craniosynostosis on computed tomography, providing objective evidence supporting a syndromic diagnosis.

Table 3 summarizes previously reported cases of GOSHS with documented neonatal presentation, including age at initial gastrointestinal symptoms, age at diagnosis, key neonatal features, and molecular confirmation status [1,3-5,7-10,13]. This table provides a structured overview of previously reported cases and highlights the variability in clinical presentation and timing of diagnosis.

**Table 3:** Reported cases of GOSHS with neonatal presentation and molecular confirmation Age at diagnosis refers to the time of clinical or molecular confirmation based on available reports. Earlier cases predate the identification of the KIFBP gene and therefore lack molecular data. GI: gastrointestinal; KIFBP: Kinesin Family Binding Protein; GOSHS: Goldberg-Shprintzen megacolon syndrome

Author, year	No. of patients	Hirschsprung disease	Age at first GI symptoms	Age at diagnosis	Molecular confirmation (KIFBP variant)	Reported neonatal features
Goldberg and Shprintzen, 1981 [1]	2 (siblings)	Yes (short-segment)	Neonatal intestinal obstruction (timing not specified)	Infancy	Not available (pre-molecular era)	Delayed passage of meconium, megacolon requiring surgery, microcephaly, cleft palate, facial dysmorphism
Hurst et al., 1988 [3]	2	Yes	Neonatal bowel obstruction (timing not specified)	Later infancy	Not available (pre-molecular era)	Microcephaly, iris coloboma, developmental delay
Yomo et al., 1991 [4]	2 (siblings)	Yes	Early neonatal obstruction	Infancy	Not available (pre-molecular era)	Hypotonia, ptosis, microcephaly
Ohnuma et al., 1997 [5]	1	Yes	Neonatal Hirschsprung disease	Infancy	Not available (pre-molecular era)	Microcephaly, central nervous system structural abnormalities
Brooks et al., 1999 [7]	3/4 affected	Yes	Neonatal Hirschsprung disease requiring early surgery	Childhood	No molecular confirmation reported	Microcephaly, craniofacial dysmorphism
Shahar and Shinawi, 2003 [8]	2 (phenotypic GOSHS within series)	Yes	Neonatal Hirschsprung disease requiring early surgery in both cases	Infancy/childhood (clinical diagnosis)	Not available (pre-KIFBP era)	Microcephaly, facial dysmorphism; agenesis of corpus callosum (1 case)
Murphy et al., 2006 [9]	2 (brothers)	Yes	Neonatal obstruction	Infancy	Not specified	Hypotonia, dysmorphic facial features
Dafsari et al., 2015 [10]	1	Yes	Neonatal Hirschsprung disease (history reported)	7 years (molecular confirmation)	Biallelic pathogenic KIFBP variant (loss of function)	Microcephaly, later sensory-motor axonal neuropathy
Rabadia et al., 2024 [13]	1	Suspected phenotype	Delayed meconium; abdominal distension at day 15	40 days	Pathogenic KIFBP variant (molecularly confirmed)	Severe constipation, feeding intolerance, microcephaly, dysmorphism
Present case	1	Yes (biopsy confirmed)	Day 4	Day 24 (neonatal period)	Compound heterozygous pathogenic KIFBP variants (segregation confirmed in trans)	Microcephaly, hypotonia, craniosynostosis, corpus callosum hypoplasia

Neonatal presentation in the literature

Neonatal diagnosis of GOSHS is distinctly uncommon. Review of the literature indicates that only a small minority of reported cases were recognized during the neonatal period, with most diagnoses made in late infancy or childhood [1-10]. Even among patients presenting neonatally with intestinal obstruction, the diagnosis is typically limited to isolated Hirschsprung disease [1,3-7,14].

Based on the available literature, cases of GOSHS diagnosed during the neonatal period appear to be exceedingly rare, with the majority of cases diagnosed later in infancy or early childhood. The typical age at diagnosis ranges from infancy to early childhood, often delayed, following the onset of developmental delay, seizures, or progressive neurological impairment [3-7,9,10,14] (Table 4).

**Table 4:** Timing of diagnosis in GOSHS based on published literature Frequency categories are based on the qualitative synthesis of published case reports and case series. Due to the rarity of GOSHS and variability in reporting, precise proportions are not consistently available. GOSHS: Goldberg-Shprintzen megacolon syndrome

Timing of diagnosis	Frequency (qualitative estimate)	Typical diagnostic trigger
Neonatal (≤28 days)	Rare	Hirschsprung disease presenting with failure to pass meconium or intestinal obstruction, often in association with hypotonia or dysmorphic features; however, syndromic recognition may be delayed at this stage
Early infancy (1-12 months)	Common	Persistent constipation and feeding difficulties combined with hypotonia; emerging dysmorphic features and neurodevelopmental concerns
Childhood (>1 year)	Common	Developmental delay or intellectual disability with or without epilepsy; neuroimaging abnormalities prompting syndromic evaluation

Compared to previously reported neonatal cases, our patient was diagnosed early in the neonatal period, with molecular confirmation supported by familial genetic findings, emphasizing the importance of early recognition in clinically suggestive presentations.

Delayed diagnosis may be associated with prolonged diagnostic evaluation, delayed neurodevelopmental intervention, recurrent hospitalizations for feeding and gastrointestinal complications, and lost opportunities for early genetic counseling and family planning.

A review of published case reports indicates that only a very small minority of reported patients have been formally diagnosed with GOSHS during the neonatal period. Notable neonatal cases from the literature illustrate the early clinical spectrum.

Shahar and Shinawi reported in 2003 one of the earliest neonatal presentations consistent with GOSHS within a broader series of neurocristopathies. They described a neonate with dysmorphic facial features, bilious vomiting, abdominal distension, and lethargy during the first week of life. Brain imaging revealed agenesis of the corpus callosum, ventriculomegaly, and cortical dysplasia. Hirschsprung disease was histologically confirmed by rectal biopsy. The diagnosis was established clinically and phenotypically, based on the combination of congenital Hirschsprung disease and characteristic dysmorphic features. This case was described in the context of a broader neurocristopathy spectrum, and molecular confirmation was not available at that time, as the KIFBP gene had not yet been identified [8].

Rabadia et al. in 2024 reported a 40-day-old male infant with progressive abdominal distension, constipation since birth, dysmorphic facial features, and persistent respiratory compromise. Whole exome sequencing identified a homozygous likely pathogenic KIFBP variant, establishing the diagnosis at 40 days of age [13].

The majority of published GOSHS cases have been diagnosed in childhood, with main manifestations including global developmental delay, intellectual disability, characteristic craniofacial features, seizures, peripheral axonal neuropathy, and cortical malformations such as polymicrogyria during neuroimaging evaluation, features which are well documented in older children and adolescents [3-9,14].

Orphanet registry data confirm that the onset of Hirschsprung disease and microcephaly often occurs in the neonatal period, but in the majority of cases, the diagnosis is delayed due to phenotypic variability and may coincide with isolated Hirschsprung disease [11].

Our case is notable for early syndromic recognition and molecular confirmation in the neonatal period, which differs from the typical diagnostic pattern reported in the literature, highlighting the importance of early clinical awareness in neonates presenting with Hirschsprung disease, hypotonia, and dysmorphic features.

Genotype-phenotype correlations

Most reported pathogenic variants in KIFBP are truncating (nonsense or frameshift), consistent with loss of function as the primary disease mechanism. In the present case, two truncating variants (c.1176_1177del, p.Glu394Thrfs*11 and c.1535C>G, p.Ser512*) were identified in compound heterozygosity. Familial genetic analysis confirmed the segregation of the variants in trans, providing strong molecular evidence for autosomal recessive inheritance and confirming the diagnosis [2,7,12].

Emerging genotype-phenotype correlations suggest that complete loss of KIFBP expression is associated with more severe neurological involvement, while hypomorphic variants may result in milder cognitive impairment [7,12]. The presence of generalized hypotonia in this neonate may be explained by impaired neuronal migration and axonal transport resulting from KIFBP loss of function. However, Hirschsprung disease appears to be a consistent feature regardless of variant type [1-7].

Table 5 summarizes reported genotype-phenotype correlations associated with KIFBP variants, highlighting the relationship between variant class, functional effect, enteric nervous system involvement, central nervous system severity, and peripheral neuropathy [2,7,12].

**Table 5:** Genotype-phenotype correlations in GOSHS associated with KIFBP variants KIFBP: Kinesin Family Binding Protein; GOSHS: Goldberg-Shprintzen megacolon syndrome

Variant type	Zygosity	Effect on KIFBP	Enteric nervous system involvement	Central nervous system severity	Peripheral neuropathy
Nonsense	Homozygous	Complete loss of function (truncating variant)	Severe Hirschsprung disease	Severe (microcephaly, structural brain abnormalities)	Reported in several cases
Frameshift	Homozygous	Complete loss of function (frameshift truncation)	Severe Hirschsprung disease	Severe	Variable expression
Compound heterozygous (truncating variants)	Compound heterozygous	Markedly reduced or absent protein expression	Severe Hirschsprung disease	Moderate to severe	Reported in recent cases
Missense (rare)	Homozygous	Partial loss of function	Variable severity	Mild to moderate	Not well defined

Clinical significance of early diagnosis

This report highlights the molecular confirmation of GOSHS in the neonatal period, supported by concordant pathogenic variants identified in an affected sibling, thereby reinforcing autosomal recessive inheritance within the family. The diagnosis was established during the neonatal period, before the development of more overt neurological manifestations typically recognized later in infancy or childhood. This case adds to the existing literature on the timing of diagnosis and supports the feasibility of early molecular confirmation when Hirschsprung disease coexists with hypotonia, dysmorphic features, or neuroimaging abnormalities. Early molecular confirmation facilitates anticipatory multidisciplinary management, structured neurodevelopmental follow-up, and timely genetic counseling for affected families.

Diagnostic implications

This case highlights several key considerations for clinicians: First, Hirschsprung disease in the neonatal period should prompt evaluation for syndromic causes when associated with hypotonia, craniofacial dysmorphism, or neuroimaging abnormalities. Next, early genetic testing, including targeted gene analysis or next-generation sequencing approaches, should be considered to support diagnosis and reduce diagnostic delay. Lastly, recognition of GOSHS in the neonatal period enables anticipatory multidisciplinary management including surgical, neurological, and genetic counseling interventions.

## Conclusions

GOSHS is a rare but clinically significant cause of syndromic Hirschsprung disease. Although congenital in origin, the condition is infrequently diagnosed in the neonatal period, often resulting in delayed diagnosis.

This case illustrates the possibility of neonatal diagnosis, supported by the molecular identification of compound heterozygous pathogenic variants in the KIFBP gene using targeted PCR amplification followed by Sanger sequencing. Increased awareness of early clinical signs, combined with appropriately timed genetic testing, may facilitate earlier diagnosis, inform clinical management, and support genetic counseling for affected families.

GOSHS should be considered in neonates presenting with Hirschsprung disease in combination with neurological signs or dysmorphic features.
